# Educational needs for medication safety competence among nurses by clinical ladder stage

**DOI:** 10.1371/journal.pone.0319483

**Published:** 2025-04-16

**Authors:** Sua Jung, Jinkyung Park

**Affiliations:** 1 Department of Nursing, Nambu University, Gwangju, Republic of Korea; 2 College of Nursing, Chonnam National University, Gwangju, Republic of Korea; School of Nursing Sao Joao de Deus, Evora University, PORTUGAL

## Abstract

The objective of this study is to identify the educational needs of nurses in regard to medication safety competence at different career levels. The identification of these needs is grounded in nurse clinical career ladder theoretical frameworks. In April of 2022, focus group interviews were conducted with twenty participants currently engaged in patient care work. These participants were categorized into four groups based on their clinical experience levels: novice (entry to 1 year), advanced novice (from 13 months to 47 months), competent (48 months to 83 months), and proficient (from 84 months). Key educational needs included: self-reflective attitude toward medication safety, effective interaction skills, medication practice knowledge, ability to manage safety risk situations, and establishment of a continuous education system for medication safety. These findings will contribute to the development of medication safety education programs based on a clinical ladder system including attitudes, knowledge, and skills related to self-reflection, self-improvement, and collaboration.

## Introduction

Medication errors cause significant harm to healthcare systems worldwide, with an estimated $42 billion in annual medical costs [1]. Medication safety is defined as freedom from preventable harm during the medication use process [2]. The most important management implication of medication errors is that most are preventable patient safety incidents [3], and medication errors are most frequently associated with serious patient harm [4].

Nurses are the most important healthcare professionals regarding medication safety [5]. The most effective strategy for preventing medication errors is empowering nurses to ensure medication safety [6,7]. Nurses have seven responsibilities in safe medication management: medication monitoring, side-effect management, medication adherence, patient self-management of medications, patient education and information about medications, prescribing, and transition of care coordination [8]. Several reviews on medication safety in nursing have explored special circumstances, such as transitional or acute care for the elderly, or registered nurses’ medication administration techniques [9–11].

Nurses’ professional competence is defined as the ability to use a set of knowledge, skills, and attitudes to successfully perform a job, role, or responsibility [12]. The proficiency model by Benner [13] is a widely accepted framework in nursing education and practice and proposes that the process occurs over time through five distinct stages of proficiency: novice, advanced novice, competent, proficient, and expert [13]. Additionally, Buchan [14] developed a clinical ladder to help nurses develop their competence and careers in the clinical field and increase their professional responsibility and influence within the organization. Similarly, Jang [15] categorized nurses into four stages of clinical experience. To develop competent nurses, education should align with their clinical careers, and practice education should match the educational needs of each clinical level.

Medication safety is currently at a high level in international and national agendas, and education is an identified strategy for addressing related issues [16–18]. Regular training and evaluation are vital for improving nurses’ medication safety capabilities [19,20]. The need for regular education and the evaluation of medication safety for healthcare providers at the national level has been proposed [1]. This study aimed to identify the educational needs of nurses at each clinical career stage with the goal of developing a systematic educational model to improve medication safety competence in the clinical field.

## Method

### Study design

This study employed a qualitative research design and focus group interviews to investigate the continuing educational needs of clinical nurses. The research question is “What is your educational needs for medication safety?” The focus group method enabled us to gain a comprehensive understanding of participants’ attitudes, opinions, beliefs, and views. Additionally, this methodology allowed us to acquire an in-depth understanding of the subject and clarify the significance of participants’ perspectives [21–23]. The process was assessed using the consolidated criteria for reporting qualitative research [24].

### Participants

The study participants were recruited between March 1 and March 31, 2022 and were classified using the clinical ladder system [15] according to their clinical career stage: novice (entry to 1 year), advanced novice (13–47 months), competent (48–83 months), and proficient (from 84 months). The inclusion criteria were as follows: 1) nurses who worked at a tertiary university hospital with a bed capacity of over 300, and 2) nurses who engaged in patient care for a period of at least three months. The exclusion criteria included nurses who were currently not engaged in direct patient care at the time of the study. Focus group interviews to derive educational needs were effectively accessed through dynamic communication between five nurses from each group.

### Data collection

Focus groups were conducted in April 2022 by using the same method to ensure consistency in data collection; a semi-structured interview guide was used to ensure similar key topic discussions by each group. The principal investigator prepared questions for the semi-structured interviews by composing introductory, transitional, main, and closing questions according to the method of Krueger and Casey [25]. All participants completed a demographic questionnaire. The interviews were conducted in a university classroom with closed doors to ensure privacy, with 80–110 min sessions. All interviews were recorded using audio equipment and a validated focus group interview guide.

All focus groups were moderated by the principal investigator. The other researchers observed the atmosphere of the field and nonverbal expressions of the subjects during the focus group and recorded them in the field notes. The research assistant recorded the meeting notes during the interview. Both the moderator and assistants took field notes during the interviews.

### Data analysis

Focus group evaluation was continuously conducted throughout the interview, and the contents of a discussion were occasionally summarized; data in handouts and tape recorders were reflected in the evaluation. After the group interview, the recording was immediately transcribed, and memos were organized.

The recorded transcripts of the focus group interviews were systematically partitioned, labeled, sequenced, and categorized using Excel. The analytical process for qualitative content was used by the first and corresponding authors to analyze the data [26]. The two researchers independently read the transcripts, identified concepts of interest by highlighting similar texts, and separately identified broad themes and subtopics in each transcript. This continued until data saturation was reached and no new subjects appeared. Finally, the two researchers compared their findings and constructed a thematic framework based on common agreement.

To obtain reliable results, the researchers repeatedly analyzed and validated the subject classifications. The researchers also retained information and left review clues to increase study reliability [27,28]. The data analysis established the objective and scientific aspects of the process by iteratively comparing the categorization of registered and categorical data (S1 Table).

### Ethical considerations

This study was approved by the Chonnam national university hospital institutional review board (IRB number: CNUH-2022–044). Participation in each focus group was voluntary, and data were reported anonymously. Written consent was obtained after explaining the purpose and scope of the study to the participants. Participants were informed that the interviews would be recorded, that the collected data would not be disclosed to third parties, and that they had the right to withdraw from the study.

## Results

Focus group interviews were conducted to comprehensively assess the need for medication safety education across various career stages in medical institutions. Twenty participants were included in the study, with five participants in each of the four career stage groups. Table 1 shows the demographic characteristics of the participants. We identified the following as crucial educational needs for medication safety: self-reflective attitude toward medication safety, effective interaction skills, medication practice knowledge, ability to manage safety risk situations, and establishment of a continuous education system for medication safety (Table 2).

**Table 1:** Demographic characteristics of the participants.

		FG 1 – NS	FG 2 – ANS	FG 3 – CS	FG 4 – PS
N		5	5	5	5
Age (mean)		23.8	25.0	27.2	37.2
Marital status	Married	0	0	0	3
	Unmarried	5	5	5	2
Education	Bachelor’s degree	5	5	5	2
	≥Master’s degree	0	0	0	3
Department	*Ward*	3	4	4	2
(Work unit)	*ICU*	2	1	1	2
	*Other*				1
Clinical career (mean)		0 yr 7 mo	2 yr	5 yr 1 mo	15 yr 7 mo
Current workplace career (mean)		0 yr 7 mo	1 yr 11 mo	4 yr	3 yr 9 mo
Medication safety education	Yes	4	2	3	3
	No	1	3	2	2

FG, Focus Group; NS, Novice stage; ANS, Advanced novice stage; CS, Competent stage; PS, Proficient stage; mo, months; y, years

**Table 2:** Themes and subthemes of educational needs for enhancing medication safety competence.

Themes	Subthemes
Self-reflective attitude toward medication safety	Attitude toward taking the principles of dosage seriously
	Attitude toward reducing medication errors
	Attitude toward voluntary reporting
Effective interaction skills	Interprofessional communication skills to prevent medication errors
	Effective teaching skills
Medication practice knowledge	Ability to find medication information independently
	Knowledge of medication administration
Ability to manage safety risk situations	Providing strategies for managing safety risk situation
	Willingness to adopt new strategies
Establishment of a continuous education system for medication safety	Increasing the quantity and quality of medication safety education
	Establishment of a career ladder education system

### Theme 1: Self-reflective attitude toward medication safety

This theme focused on fostering self-reflective attitudes toward medication safety among healthcare professionals. Most participants acknowledged that medication safety incidents and near-miss errors were often due to non-adherence to the fundamental principles of the “five rights of medication administration.” Incidents were associated with busy work environments, where factors such as improper patient identification and administration of frequently used drugs or medications with similar packaging increased the risk of medication safety incidents.

“It is really important to get into the habit of thoroughly checking patient information. I once had a patient with the same name and age as another patient, that belonged to a different department. Although I had checked their names, I did not verify their ID number. It turned out that the ID number did not match, and I realized that I needed to ensure I always check the ID number along with the patient’s name and other details to avoid any mix-ups.” (FG 4, P 1)

Groups 3 and 4 displayed a heightened sense of awareness toward medication safety and emphasized the need for education. Participants in less experienced groups had a lower awareness of medication safety, repeated errors, and made less effort to reduce errors.

“I feel like new nurses these days are not alert at all because they make the same mistakes repeatedly. They do not react at all, even when I address them, because it is obviously something that could be a big deal. The other day I saw a new nurse giving a patient a pill that had fallen out of his mouth. The patient said that it had fallen out, and while new medication should be administered, it was not.” (FG 4, P 1)

There was also no environment for the voluntary and immediate reporting of errors; therefore, a culture that encourages voluntary reporting of all medication errors, including near-misses, is needed to prevent the recurrence of medication errors. In Group 4, the voluntary reporting of medication errors is not encouraged because managers tend to focus on problem resolution and individual accountability rather than on providing constructive feedback.

“When I see people writing medication error reports in the ward, it is not like they are writing a report, but a letter of apology. Therefore, it is kind of a criminal offence, and I think it is a big thing to ask nurses to write a medication error report, more so than in other departments.” (FG 2, P 1)

Therefore, education on medication safety should be implemented to address attitudes that disregard the principles of medication administration, demonstrate reduced concern for medication safety, and exhibit hesitance to voluntarily reporting medication safety incidents.

### Theme 2: Effective interaction skills

Education on communication skills is important to enhance cooperation and teamwork among medical personnel. Insufficient communication between senior and junior nurses often leads to duplications in medication preparations. Furthermore, some medication errors were due to junior nurses’ reluctance to seek advice from senior nurses regarding administrative procedures. Miscommunication between physicians and nurses regarding medication prescriptions can also lead to errors, with verbal prescriptions being the most common cause. A participant in Group 2 said, “The doctor asked me to use only half an ampoule, which I complied with. However, I received a phone call asking why only half the ampoules were used.” Novice nurses faced communication challenges with physicians when verifying medication prescriptions. Therefore, effective interdisciplinary peer-to-peer communication skills should be developed to ensure safety.

“In the past, even if I was scolded by a senior colleague, I would ask everything, and if there was a change, I would ask the senior and the doctor; now, they do not ask the senior colleague and doctor. They call and report to the doctor separately in the changing room so that the seniors outside cannot hear. I heard that they have their own peer chatrooms, and if they do not know something, they ask each other. They do not ask their seniors; they just enquire amongst themselves. I do not really understand that either.” (FG 4, P 4)

Participants with more clinical experience indicated the need for effectively educating others, highlighted the inadequacy of medication education, and emphasized the importance of instilling proper medication habits among patients. The training of junior nurses by senior nurses is also ineffective, thus highlighting the need for the effective training of junior nurses in the clinical field.

“Sometimes there is a medication on a patients’ prescription list that they must take regularly by themselves at a certain time. If the dose of that medication changes during the hospital stay, we tell the patient, and they say they have already taken it at the original dose, or complain that we do not let them take it when we try to educate them.” (FG 3, P 1)

### Theme 3: Medication practice knowledge

All groups consistently expressed the need for medication education. They highlighted their limited knowledge of medicine effects, usage, side effects, and mechanisms and indicated the need to learn more about these topics on their own. Participants believed that reliable medication information about medication actions would improve their understanding of newly introduced or rarely used medications. Nurses should independently search for medication information because education may not always be available to them. If medication safety education includes information on medication actions, nurses would be able to easily understand the medication information for new drugs.

“In my workplace, which is an intensive care unit, we frequently receive patients from different departments that are not under our primary area of responsibility. Sometimes, I encounter unfamiliar drugs that I have not administered before, and I am directed to use them as prescribed without receiving prior instructions or training on their usage.” (FG 1, P 4)

Less experienced participants rarely identified prescription errors and focused on specific situations without considering the entire treatment process; additionally, less experienced nurses struggled with dosage calculations. Groups 1 and 2 requested education on reviewing and administering prescriptions to improve medication safety.

“A junior nurse administered Lasix to a patient who required a reduction in their fluid volume. Within 1–2 hours, the patient’s volume had decreased by 4000 ml, and their blood pressure had slightly decreased. However, she only reported the blood pressure drop to the on-duty doctor, who was not familiar with the patient’s medical history. Consequently, the doctor ordered a loading dose, leading to a vicious cycle. To prevent such incidents in the future, I was instructed to take detailed notes and observe the patient closely.” (FG 4, P 4)

### Theme 4: Ability to manage safety risk situations

Given that the participants were aware of the response guidelines to medication errors, they panicked and were unable to respond appropriately. Reporting errors and investigating the root causes in these risk situations enables the implementation of preventive strategies. Nevertheless, all groups had limited knowledge about the root causes of medication errors, strategies to prevent recurrence, and tools such as Root Cause Analysis (RCA) and Failure Mode Effectiveness Analysis.

“During my medication safety education, I analyzed the cases but could not get to the root causes.” (FG 3, P 2)

If a new strategy to prevent medication error recurrence is proposed, it should be applied equally to all departments in the hospital and followed by all staff; however, participants in all groups reported that new processes or equipment was often not used because existing practices and instructions differed between wards. Efforts to standardize feedback on medication errors were minimal, and feedback varied between wards, thus leading to confusion.

“In our ward, a particular medicine is labeled with shading, whereas in other wards, it is not. Recently, a patient from another ward asked me about this difference and which approach was correct. However, as we are all part of the same hospital, it was difficult for me to provide a clear answer regarding what was right or wrong.” (FG 2, P 1)

### Theme 5: Establishment of a continuous education system for medication safety

Participants with more experience requested detailed analyses of medication error cases, whereas Group 2 requested examples. All groups acknowledged that medication safety education was insufficient and raised concerns about the limited opportunities for face-to-face education owing to varying work shifts and inconsistent end times. Even when changing wards, there were few educational opportunities to adapt to the new working environment.

“I did receive medication safety education during the new employee training, but I found the level and content to be quite basic, almost at an elementary school level. It was rather ordinary and unremarkable.” (FG 2, P 3)

Experienced individuals described the implementation of a clinical ladder system with tailored educational opportunities for maintaining learning motivation. Beyond their on-the-job training, the participants received minimal training in medication safety. Groups 2 and 3 recognized the potential for errors due to poor adherence to medication practices and highlighted the importance of ongoing education and correcting such behaviors.

“Yes, they were careful for a bit, but it does not last. That is why I think it is important to keep learning and educating yourself.” (FG 4, P 5)

“We teach emergency medications when you are new, but there are things that you learn through experience. While charge nurses like us know that, less experienced nurses are confused about medications that they do not use often. More experienced nurses are able to work with the overall condition of the patient, and less experienced nurses cannot do that. They need training in medication administration, and they are less vigilant than we are and need training in that area.” (FG 4, P 3)

“Medication safety education is somewhat included in the onboarding training. But it is not really education, it is more like an extension of the basic content that even elementary school students would take for granted. It is like education, that...is at the school level. It does not really suit us...” (FG 2, P 3)

## Discussion

This study examined nurses’ educational needs to improve their medication safety competencies through focus group interviews and was conducted among four career stage groups, thus maintaining the homogeneity of the focus groups and allowing for the identification of educational needs at each career stage. Five themes were identified regarding nurses’ educational needs for the improvement of medication safety competence. First, nurses may become careless and disregard established principles (e.g., the five rights of medication administration) because of their familiarity with the routine. Therefore, education that encourages self-reflection and vigilance for medication safety is needed to prevent complacency and carelessness [29]. Additionally, participants highlighted the importance of maintaining attention and vigilance regarding medication safety by minimizing distractions and focusing on high-risk medications during medication administration [30].

The responsibility of nurses in medication safety competence is emphasized, and the need for educational efforts to promote a continuous, reflective attitude toward medication administration and help nurses develop as experts is highlighted [7]. The usefulness of self-reflection as a tool for improving the effectiveness of educational interventions aimed at strengthening medication safety competence in nursing education is also highlighted [31]. Practices such as maintaining a journal or integrating open-ended questions can effectively reinforce self-reflective attitudes [32,33].

Reporting medication errors is crucial to ensure patient safety and prevent safety issues [34]. The Patient Safety Act, which was enacted in Korea in 2016, established a system to autonomously report and learn from medication errors [35,36]. Nevertheless, medication error reporting in South Korea remains inadequate and requires further research for improvement [37,38]. The participants reported that they were discouraged from voluntarily reporting medication errors because of cumbersome reporting procedures, fear of disclosing their identity, fear of being held accountable for patient deterioration, an atmosphere of blame, and coercion. This finding corresponds with the barriers to voluntary reporting identified in previous studies [39,40]. Encouraging the voluntary reporting of medication errors requires the implementation of reporting systems that provide anonymity, incentives for reporting, expert analysis and response, training on the use of the reporting system, and assurance that reporting medication errors is not punitive but is the first step in improving the patient safety environment [38,41].

Effective interaction skills were the second most critical educational need for improving medication safety competence among nurses with different experience levels. Experienced nurses highlighted that less experienced nurses tended to hesitate seeking guidance from their senior colleagues when encountering unfamiliar situations. They also expressed uncertainty about the most effective approach to educating less experienced nurses. In today’s workforce, which is characterized by tech-savvy and direct generation Z nurses who value personal interaction, adapting communication methods is crucial to fostering efficient teamwork [42]. Incorporating Situation–Background–Assessment–Recommendation training, which has a positive impact on interprofessional and intrateam communication and significantly contributes to patient safety, into medication safety competence education can increase nurses’ communication skills and improve teamwork in clinical settings [43,44].

Experienced nurses expressed the need for specific techniques to effectively educate patients on the process of medication administration. Patient education is a low-tech solution for ensuring medication safety [30], and effective communication and education about medication are essential [7]. Nurses must supervise and guide patients to ensure compliance with correct medication regimens. Lectures, group discussions, reflections, feedback, and role-playing [45] can improve effective communication skills with patients. Therefore, a curriculum is needed to improve the competence of nurses through these various methods.

Third, participants emphasized that knowledge beyond medications is needed. Although nurses require competencies such as in pharmacology and medication calculation [46], the current study emphasizes the need for develop self-learning skills. Considering that nurses frequently encounter new medications, the continuous acquisition of medication knowledge is vital. Hence, educational interventions should not be limited to basic medication information but should also include the capability to learn independently based on an understanding of pharmacological actions. Furthermore, nurses should manage the medication process by assessing patients and situations, planning and administering medications, evaluating patient responses, and conducting medication education [5,10]. Therefore, nurses should have holistic knowledge of the medication process. As demonstrated by the studies conducted by Mandani et al. [10] and Park and Seomun [7], nurses should play a central role in multidisciplinary cooperation for medication safety.

Park and Seomun [7] presented the “improvement of safety problems” as a medication safety competence, which refers to the competence to identify the root cause and establish appropriate measures to prevent similar incidents. Fourth, participants in this study expressed that they were unfamiliar with RCA tools and lacked the experience to analyze the root causes of medication safety incidents. Given that RCA is widely used to prevent medical errors [47], RCA training for healthcare professionals is essential to improve medication safety and bridge the gap between incident analysis and educational initiatives. Simulation-based, case-based, and flipped learning methods can be used to improve RCA skills [48,49]. All relevant healthcare staff, including nurses, should be included in the RCA process.

Finally, all participants emphasized the need for ongoing medication safety education, especially at different stages of their career. Continuous education and evaluation is important to improve nurses’ medication safety competencies [50,51], and healthcare providers should undergo regular education and evaluation for medication safety at the national and institutional levels [1].

This study explored the educational needs of nurses working in clinical settings and compared previous research that focused on analyzing nursing education for medication safety [52]. The current study suggests that clinical experience should be the basis for a continuing education system. Participants also identified different educational needs based on their clinical experience [53]. To improve medication safety competence, education needs to focus on groups and organizations, foundational knowledge, and self-reflective and developmental attitudes. Group 1 underscored the significance of improving interprofessional communication skills and knowledge of medications and prescriptions. The educational needs in Group 2 were similar to those of Group 1, with a focus on intrateam communication and the importance of ongoing educational programs. Groups 3 and 4 indicated that education should address the root causes of medication safety incidents, and Group 4 emphasized the importance of developing self-reflective attitudes.

A curriculum based on a clinical ladder system is effective regarding satisfaction, motivation, and competence development [54]. Continuing education is essential for professional competence development [55]. Therefore, systematic educational programs should be designed to ensure that appropriate education is continuously provided to nurses with different levels of clinical experience to improve medication safety. During their continuing professional development, nurses reported facing environmental barriers, such as limited accessibility to resources and inadequate support [56]. The use of appropriate incentives for participation and the creation of an organizational atmosphere, along with adequate economic support, could facilitate the establishment of a clinical ladder education system [57].

The present study’s limitations are twofold: first, its focus on nurses from a single type of tertiary hospital in one country; and second, its restriction to a specific sample. Educational requirements for improving medication safety competencies may differ depending on the clinical setting. Nevertheless, our study can serve as a foundation for the development of customized educational programs for different career stages, and our findings may be relevant in other clinical settings.

## Conclusion

Collaboration among nurses with varying experience is a defining aspect of nursing tasks, and a mutual understanding of the competencies required at different levels of experience is essential. Recognizing and complementing each other’s deficiencies in competence levels can lead to the development of collaborative skills. Our results highlight specific educational aspects vital to promoting medication safety competencies among healthcare professionals. Recognizing the differences in educational needs across career stages can enhance teamwork, and healthcare institutions can customize their educational programs to effectively address the unique requirements of each group. This approach will significantly promote safety and optimize patient outcomes.

## Supporting information

S1 Table Code, subtheme, and theme mapping from interview data. (PDF)
