# Supplementary material for: Educational needs for medication safety competence among nurses by clinical ladder stage
Source: PLoS One. 2025 Apr 16;20(4):e0319483. doi: 10.1371/journal.pone.0319483 (PMC12002428; doi:10.1371/journal.pone.0319483)
Supplement: S1 Table — (PDF) [file pone.0319483.s001.pdf]

S1 Table. Code, Subtheme, and Theme Mapping from Interview Data

	coding (concept)	grouping	sub theme	theme
G4	Medication errors occur when patient identification is not properly verified during busy times.	Errors occur when patient identification is not performed according to the proper procedure.	Attitude towards taking the principles of dosage seriously	**Self-reflective attitude toward medication safety**
G4	There are no medication errors in the outpatient injection room because patient identification is thoroughly verified.			
G4	The habit of verifying patient identification is important.			
G4	A near-miss error occurred due to the failure to develop the habit of properly verifying patient identification.			
G4	The method of patient verification to prevent medication errors is not becoming a habitual practice.			
G4	New nurses tend to become careless with patient verification once they become somewhat familiar with their tasks.			
G4	Administering another patient's antibiotic to a patient.			
G3	Medication errors occur when patients are remembered by their bed number instead of their name, leading to mistakes when patients are moved to different beds.			
G3	Medication errors occur when there are multiple patients receiving chemotherapy on the same cycle, or when patients have similar names, especially when two or more chemotherapy patients are in the same room.			
G3	Medication errors may occur if open-ended questions are not asked properly, and if the patient's incorrect response is not re-verified.			
G1	The most important aspect of medication safety is patient identification.			

G3	In busy situations, patient verification is often overlooked when a verbal order is given.	In busy situations, patient verification is not properly performed.
G3	Medication errors occur when frequently used painkillers are administered without a medication card, especially when mixed and administered in a busy situation.	In busy situations, the ward protocol is not properly followed.
G3	Medication errors occur when discharge medications are not checked due to a busy workload, leading to incorrect dosage instructions being given.	
G1	Medication errors occur when a different medication is used in haste.	
G1	A near-miss error occurs when the hospital ward processes are not followed.	
G4	A medication error occurred due to an incorrect dosage, resulting in an overdose of IV fluids.	A medication error occurred because the proper procedure for verifying medications was not followed.
G4	A medication error occurred due to confusion between similar containers, leading to incorrect administration.	
G4	A medication error occurred due to the incorrect administration of a sedative with a similar name.	
G4	A medication error occurred due to the incorrect dosage being administered.	
G3	A potential medication error was prevented by checking the remaining product after confusing similar items, thus avoiding a near-miss error.	
G3	A near-miss error occurred in the disorganized medication preparation room due to confusion between similar products.	
G2	A medication error occurred when a medication with the same cap color was confused and administered without additional verification.	
G2	A medication error occurred when the mixing label was not checked, leading to the use of the wrong IV fluid.	

G2	A medication error occurred when the heparin sticker was only checked, and the correct order was not verified before administration.		
G1	It is important to verify the dosage when administering sedatives.		
G1	Caution should be taken when using medications stored in similar containers to avoid confusion.		
G1	A medication error occurred when chemotherapy was administered without knowing the proper administration method.		
G1	A medication error occurred due to incorrect dosage verification following a change in the barcode format.		
G1	In the case of an incident involving controlled substances, the complex reporting procedure may lead to failure in reporting the event	The controlled substances management procedure was not followed.	
G1	Due to the complex procedures for controlled substances management, the process was handled for convenience.		
G4	The problem is that the awareness and vigilance are only maintained for a short period after the incident.	Errors occur due to decreased vigilance in familiar medication practices.	Attitude toward reducing medication errors
G4	When tasks become familiar, vigilance tends to decrease.		
G4	When a medication error occurs, the person involved is required to write the report themselves to raise awareness and maintain vigilance.		
G4	When working functionally, there is a tendency to work out of habit, which can lead to a loss of vigilance and an inability to recognize mistakes.		
G4	Education is needed to raise awareness and vigilance among less experienced nurses.	Education is needed to raise awareness and vigilance among less experienced nurses.	
G4	Even when working functionally, it is important to have a sense of responsibility, remembering that the patient is your own.		

G4	Due to generational differences with new nurses, it can be challenging to deliver guidance aimed at raising their awareness and vigilance.		
G1	To prevent medication safety incidents, constant focus and attention are required.		
G3	As experience accumulates, the need for education on vigilance, awareness, and responsibility becomes more apparent.	Education on vigilance and responsibility is needed to prevent medication errors.	
G3	Education to raise awareness and vigilance regarding medication errors is essential.		
G2	As practice becomes more familiar, it is important to maintain vigilance and work with a sense of responsibility.		
G4	An attitude of not following infection control principles during medication administration without feeling guilty.	An indifferent attitude towards infection control principles.	
G4	An atmosphere that prioritizes customer satisfaction over patient safety.	Prioritizing customer satisfaction over patient safety.	
G3	A medication error occurred when other staff members, while the patient was away in the testing room, handled the pump and IV line carelessly without verifying the medications being administered.	Other healthcare professionals need to be reminded to pay attention to medication safety.	
G3	A near-miss error caused by confusing similar products was reported to a superior without concealment, contributing to process improvement without facing blame.	There is a need to create an atmosphere that encourages the voluntary reporting of near-miss and medication error incidents.	Attitude to report voluntarily
G3	In the past, near-miss and medication errors were reported to the entire ward, but the system has since changed to a culture where they are reported to the manager		
G3	An atmosphere is being created where errors are reported voluntarily rather than concealed, allowing for improvements.		
G3	When medication errors or near-miss errors are reported, the hospital conducts a situation analysis and provides guidance to prevent recurrence.		

G3	During preceptorship training, sharing personal experiences conveys a powerful message to the trainees. There is a need for a platform to share such experiences.	
G2	In the integrated nursing and caregiving ward, staff are unable to freely write medication error reports.	
G2	There is an atmosphere where writing a medication error report is seen as a serious offense by nurses.	
G2	In the past, there was an atmosphere where the person involved and the incident were publicly disclosed by name when a medication error report was written.	
G2	Due to issues with the incident reporting system, incident reports were inevitably made visible to the entire ward.	
G1	When a safety incident or near-miss occurs, it is discussed with a superior and then reported to the management level.	
G4	When medication errors occur, the blame is placed on the individual, and there is no improvement in the system.	The responsibility for medication safety incidents is shifted onto the individual.
G4	In emergency situations, over-infusion of IV fluids is a frequent occurrence, but it often goes unreported.	
G2	In an oppressive atmosphere, individuals may hesitate to report mistakes immediately.	
G4	There is no educational feedback after writing the incident report.	Medication safety incident reports should be linked to education.
G4	A manager's attitude prioritizing identifying responsibility over education for preventing recurrence.	
G4	The manager's response to the medication safety incident focuses solely on resolving the issue.	
G2	When a medication error occurs, there is no effort to improve the situation beyond reporting and scolding.	

G4	Feedback sharing on medication safety incident cases varies by ward.			
G2	There is a lack of effort to standardize medication administration methods across wards.			
G2	Confusion caused by different standardized administration methods across wards.			
G2	There is no attempt to improve the inconsistent medication administration methods across wards.			
G4	In the emergency room, communication between senior and junior staff is often poor, leading to situations where double orders are given.	Medication safety incidents occurred due to a lack of communication between senior and junior staff.	Interprofessional communication skills to prevent medication errors	**Effective interaction skills**
G2	Errors occur due to the lack of a culture of asking questions between senior and junior staff within the ward.			
G2	Medication speed control errors occurred due to missed handoffs and a lack of knowledge during the early days of employment.			
G3	There are frequent instances where communication between healthcare professionals regarding medication prescriptions is not smooth.	Medication safety incidents occur due to communication issues between healthcare professionals.		
G2	After verbal orders are given, there are instances where the prescribed medication is administered incorrectly.			
G2	Communication errors occur regarding the prescribed dosage during verbal orders.			
G1	There are communication issues with the physician when confirming medication prescriptions.			
G2	New nurses often lack sufficient communication with physicians when confirming medication prescriptions.	Education on communication between healthcare professionals is necessary.		
G3	Medication education for patients is not being effectively carried out.	Education on how to properly provide medication education to patients is necessary.	Effective teaching skills	

G3	When a patient's prescribed medication changes, their habitual use of previous medications can prevent proper medication education from being effectively delivered.			
G4	Junior nurses are often afraid to ask questions to their seniors, which leads to a reluctance to seek guidance.	Training on communication between senior and junior staff is necessary.		
G4	Due to the social issue of "burnout," it becomes difficult to provide constructive criticism or give reminders to less experienced nurses.			
G4	Due to the social issue of "burnout," effective education for junior nurses is not being properly carried out within the ward.			
G4	There is a lack of education on specific medication administration methods, such as whether the medication should be mixed or given as a bolus.	Education on the effects and administration methods of each medication is necessary.	Ability to find medication information independently	**Medication practice knowledge**
G4	There is a need for education on medications that are not classified as high-risk but have specific effects or efficacy.			
G3	Education on drug side effects and mechanisms of action is necessary for patient education.			
G2	not received education on medications (such as their effects, side effects, etc.).			
G2	Studying the medications, dosages, and patient responses for internal medicine patients, even if it's not the main department in the ward, is important for ensuring appropriate treatment and care.	Education on medications used in departments other than the main department of the ward is necessary.		
G2	It is important to study medications from other departments			
G1	It is necessary to study the main medications used in other departments.			
G2	When using new or unfamiliar medications, ask senior staff and confirm with them before administering the medication.	Education on newly used medications is necessary.		

G4	It is necessary to engage in self-learning regarding medication education.	It is necessary to fulfill the desire for self-learning about new medication knowledge.			
G3	There is a desire to study medications independently. Developing a habit of self-learning is necessary.				
G2	There is a desire to self-learn about unfamiliar medications.				
G2	The incorrect medication was hastily used without considering the excessive financial burden on the patient.	Basic education on the health insurance coverage system for medication prescriptions.	Knowledge of medication administration		
G4	Junior nurses tend to follow prescriptions without critical thinking.	Training on reviewing prescription records.			
G4	They attempt to carry out prescriptions mechanically without critical thinking, failing to catch incorrect prescriptions.				
G4	Nurses with less experience tend to make judgments and take actions based on isolated situations without considering the patient's overall condition.				
G1	In cases where a medication error involving high-risk drugs leads to a patient safety incident.				
G1	Verifing prescriptions based on the patient's condition to prevent near-miss errors.				
G2	Independently study dosage changes in internal medicine medications.	Training is needed on medication dosage calculation and adjustments for patients with internal medicine conditions.			
G2	Having difficulty calculating medication dosages in the internal medicine ward.				
G2	Issue with fine-tuning the dosage of steroid preparations.				
G3	The ward safety guardian reviews error incidents, considers prevention methods, and conducts training within the ward.	There is no root cause analysis conducted when analyzing medication safety incidents.	Increasing the quantity and quality of medication safety education	**Establishment of continuing education system for medication safety**	theme5
G4	Due to COVID-19 issues and a culture that discourages overtime work, the time for sharing medication error cases has decreased.	There is a lack of medication safety training.			

G4	Aside from the safety guardian, there are almost no activities for preventing and improving medication safety incidents.		
G3	There is no training on behaviors or situational analysis related to medication safety.		
G1	There is a lack of sufficient training period for new staff.		
G1	Reduce the burden on new nurses to decrease the occurrence of patient safety incidents.		
G1	Did not receive training on how to handle and prevent patient safety incidents.		
G2	Sharing medication error cases with narrow content, focusing primarily on patient identification.	There is a need to use a variety of cases.	
G2	Medication safety training is conducted with simplistic cases and content.		
G3	The incident of breaking an anticancer drug during my early days has been engraved in my heart, making me more cautious.		
G2	There is a need to use educational materials that create a visual imprint.	There is a need to enhance the educational effect by using visual materials.	
G2	I would like to attend the medication training that I missed due to different shift end times on the ward.	The accessibility to training sessions is limited.	
G1	I want to apply for additional training, but it is difficult due to a shortage of staff.		
G1	I want to apply for additional training, but there is no option to choose it.		
G4	Continuous training is needed to prevent repeating mistakes.	There is a lack of experience in completing medication safety training after the initial education, so continuous training is needed.	Establishment of a career ladder education system
G4	Simple staff shortages are not the sole cause of medication errors.		
G4	Due to insufficient training within the ward, there has been a lack of growth relative to experience.		

G3	Only after becoming a safety guardian following the initial training did I receive education based on medication/near-miss error incidents.				
G3	After the initial onboarding training, I did not receive proper education on high-risk medication administration (management, dosage, etc.).				
G3	After the onboarding training, medication safety education is only conducted as part of the continuing education.				
G3	After the onboarding training, medication safety education is conducted in the form of feedback on case studies.				
G3	Training is needed to help break bad habits/routines and establish proper procedures as a habit.	Correcting bad habits/routines.			
G2	An incident occurred due to the use of a commonly accepted medication administration method with an incorrectly labeled medication.				
G2	The situation was improved by correcting the medication administration instructions on the label, which led to the revision of the conventional work method.				
G4	It is important to have the determination to study and maintain a sense of vigilance.	The willingness to continue learning is important.			
G4	The willingness to learn on one's own is important.				
G4	Performing prescriptions mechanically without the willingness to study new medications.				
G3	While analyzing issues based on medication/near-miss error incidents, a root cause analysis is not conducted.	There is a lack of knowledge about root cause analysis tools such as RCA (Root Cause Analysis) and FMEA (Failure Mode and Effects Analysis).	Providing strategies for managing safety risk situation	**Ability to manage safety risk situation**	theme4
G4	Even when medication safety incidents occur, fundamental solutions are not being implemented.				

G3	When near-miss or medication errors occur, the report is not made due to the inconvenience or hassle of the process.	Lack of awareness about the importance of reporting.	
G4	Education for physicians on the importance of reducing verbal prescription incidents is also necessary.	Lack of awareness about the importance of standardization.	Willingness to adopt new strategies
G3	Healthcare professionals lack awareness about minimizing verbal prescription incidents to reduce medication errors.		
G4	The inconvenience of using devices introduced to prevent medication errors has led to a decrease in their utilization.		
G4	The system to suppress verbal prescription incidents does not align with real-world practices.		
G3	In the nurse-managed care ward, the work protocol has changed to not only distribute prescribed medications but also confirm the	Lack of willingness to adapt to new protocols.	
G3	It is difficult to establish the habit of using equipment to reduce medication errors.		
G3	When introducing new processes for preventing safety incidents, it is essential to include them in the onboarding training.		
G1	The use of machines helps reduce patient safety incidents and enhances synchronization with electronic documentation.		
G1	Due to the inconvenience of changing work methods, the use of machines is not frequently adopted.		
